# Study of the effect of exercise fatigue on the gut microbiota of boxers

**DOI:** 10.3389/fmicb.2025.1531490

**Published:** 2025-08-08

**Authors:** Su Mei-hua, Jin Jia-Hui, Ding Wen-Long, Chen Jian-Ming, Qin Yu-Fei, Chen Jian-Hua

**Affiliations:** ^1^School of Physical Education, Jimei University, Xiamen, Fujian, China; ^2^Xiamen Sports School, Xiamen, Fujian, China

**Keywords:** exercise fatigue, gut microbiota, athletes, 16S rRNA, exhausted exercise

## Abstract

**Objective:**

To explore the changes in gut microbiota in response to exercise fatigue, to provide a theoretical basis for the diagnosis and intervention of exercise fatigue.

**Methods:**

In this study, 16S rRNA sequencing of feces from 17 youth boxers was performed before and after fatigue using the Illumina HiSeq platform. After denoising and OTU clustering of the raw sequencing data using the QIIME2 DADA2 analysis pipeline, differential microbiota were analyzed using R statistical software.

**Results:**

ASV clustering analysis revealed that 2,470 Amplicon Sequence Variants (ASVs) were common between pre-and post-exercise fatigue, while 8,321 ASVs were unique to pre-exercise and 6,341 ASVs were unique to post-exercise. There was no statistically significant difference in *α*-diversity and *β*-diversity between pre-and post-exercise fatigue, although a trend of decreasing diversity was observed. At the phylum level, the relative abundance of *Firmicutes* and *Bacteroidetes* decreased, while the abundance of *Actinobacteria* and *Proteobacteria* increased. Using LEfSe multilevel species difference discriminant analysis, a total of 12 specific phyla were identified (|LDA| > 2, *p* < 0.05), with the top three phyla ranked by relative abundance being *Prevotella* (*p* = 0.025), *Corynebacterium* (*p* = 0.038), and *Lachnospira* (*p* = 0.004). The relative abundance of *Prevotella*, *Corynebacterium*, and *Lachnospiracae* increased differently in response to exercise-induced fatigue. *Prevotella*, *Corynebacteriales*, *Lachnospiraceae* were positively correlated with creatine kinase, and *Lachnospiraceae* was positively correlated with RPE scale.

**Conclusion:**

Exercise-induced fatigue may produce specific changes to the gut microbiota that correlate with exercise-induced fatigue markers.

## 1 Introduction

Exercise fatigue is defined as the inability of an organism’s physiological processes to sustain functioning at a specific level and/or to maintain a predetermined exercise intensity (Proske, 2019). It is a common physiological phenomenon, primarily caused by abnormalities in the host’s metabolism. Excessive fatigue can exacerbate metabolic disorders, affect the physical and mental health of the host, reduce immunity, lead to endocrine dysfunction, and even result in serious complications (Proske, 2019). The generation of exercise fatigue is often accompanied by disorders of energy metabolism, accumulation of metabolic products, disruption of cellular regulatory enzyme systems, and toxic effects of oxidative stress on cells (Cordeiro et al., 2017). Ultimately, it reflects the disruption of internal environmental homeostasis (Cordeiro et al., 2017). The gut microbiota, as the second largest gene pool in the human body, plays a crucial role in regulating energy metabolism and oxidative stress (Luca et al., 2019). The gut microbiota of athletes differs from that of the general healthy population. A better understanding of the gut microbiota of athletes can help assess their physical condition, allowing for targeted training and dietary regulation, which positively impacts athletic performance (Petersen et al., 2017). Studies have shown that long-term fatigue can lead to changes in gut microbiota, aggravating the imbalance of the body’s internal environment and resulting in gastrointestinal issues such as diarrhea, constipation, and dyspepsia (Dao et al., 2016). Long-term excessive and high-intensity training may exacerbate the imbalance of athletes’ intestinal environment, affecting training outcomes and competitive performance. However, there is a lack of studies on the acute effects of exercise-induced fatigue on the gut microbiota of athletes. Therefore, this study aims to further understand the occurrence of exercise fatigue and explore the potential role of gut microbiota in combating fatigue. We selected gut microbiota samples from boxing athletes, collected fecal samples before and after exercise fatigue, and performed 16S rRNA amplicon sequencing using the Illumina HiSeq platform to analyze the structure of the athletes’ gut microbiota at various taxonomic levels. This research aims to provide a reference for the development of novel anti-fatigue healthcare products and personalized microbiota interventions.

## 2 Materials and methods

### 2.1 Study design and inclusion criteria

#### 2.1.1 Inclusion criteria

① No history of special diseases; ② No smoking or drinking habits; ③ No special medications or physical discomfort before training.

#### 2.1.2 Exclusion criteria

① Subjects unable to participate in training on time due to disease or sports injury; ② Using antibiotics or suffering from chronic gastrointestinal diseases and other factors affecting gut microbiota in the last 3 months; ③ Serious mental illness; ④ Subjects unable to meet the diagnostic criteria for sports fatigue.

This study employed a before-after design within the same subjects. Thirty adolescent boxers (15 males and 15 females) were initially screened, but 13 were excluded for not meeting the criteria for exercise-induced fatigue. Ultimately, 17 adolescent boxers (9 males and 8 females) were included in this study. The entire study process was approved and supervised by the Academic and Ethics Committee of Jimei University (No: JMU202407066). All subjects voluntarily signed the informed consent form. The basic information of the subjects is shown in Table 1.

**Table 1:** Basic information table of the included subjects.

Sex	Age (years)	Height (cm)	Weight (kg)	Years of training (years)
Male	17.33 ± 0.71	170.78 ± 5.95	57.67 ± 6.1	3.44 ± 0.73
Female	17.63 ± 1.06	162.88 ± 4.67	52.63 ± 2.88	3.44 ± 0.68

### 2.2 Experimental methods and grouping

#### 2.2.1 Experimental grouping and assessment of CK and urinary protein

In this trial, subjects who met the inclusion criteria and were diagnosed with exercise fatigue had their blood, urine, and feces collected before and after exercise fatigue. Urine and blood samples were used to diagnose exercise fatigue, while fecal samples were analyzed to detect differences and changes in gut microbiota composition. Pre-exercise fatigue was designated as group Q1, whereas post-exercise fatigue was designated as group Q2. Blood samples were collected to determine creatine kinase (CK) values. The procedure was as follows: On the morning of the day of boxer training, between 6: 00 and 8:00 AM, a professional nurse conducted the first venipuncture to collect 5 mL of blood. The sample was immediately placed in an ice box and transported to the laboratory within 1 hour. It was measured using the BC300 Semi-auto Biochemistry Analyzer from CONTEC MED to determine CK levels. On the morning of the second day following training, at the same time as the first collection, a second venipuncture was performed, and the same procedure was followed to assess CK levels. Urine samples were collected to determine urinary protein levels. The procedure involved collecting midstream urine samples from athletes before and after their physical training. The Uritest200 analyzer was used to measure the urinary protein content in these samples.

#### 2.2.2 Exercise fatigue modeling

**Figure 1:**
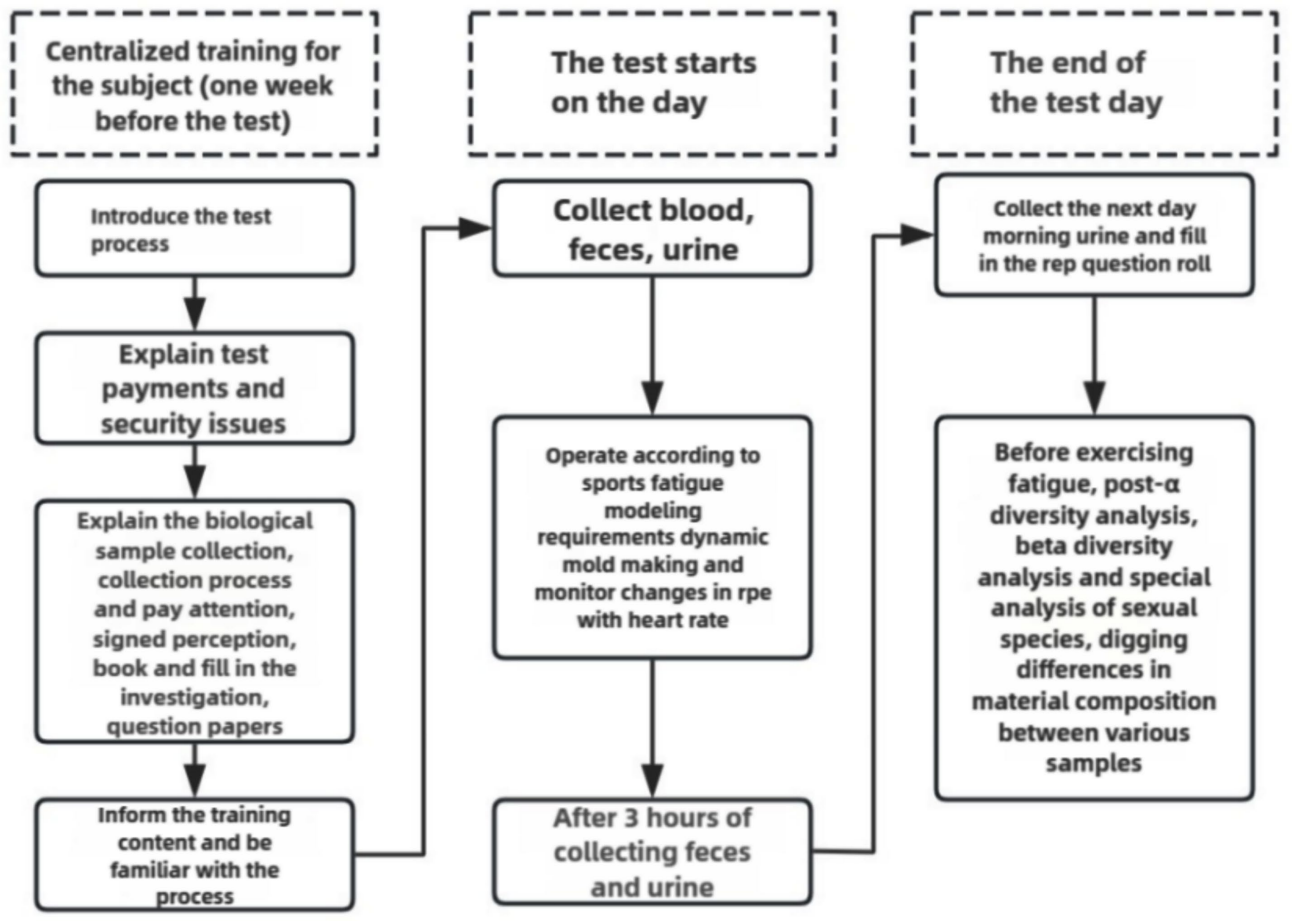
Test flow chart.

One week before the exercise fatigue modeling, systematic training was provided to all subjects, including: introduction of experimental requirements, signing of informed consent form, notification of experimental risks, and sample collection training. Subjects were asked to refrain from high-intensity and strenuous exercise for 48 h prior to the exercise modeling. All subjects were trained in accordance with the test method of Lin et al. (2009) and Duncombe et al. (2023) modified to incorporate the special characteristics of boxers, aiming to improve the training protocol. All subjects included in the study underwent step-by-step incremental high-intensity interval training as the main form of intervention, the specific experimental process is shown in Figure 1.

##### 2.2.2.1 High-intensity interval incremental training mode

All subjects were required to complete 20 min of dynamic stretching and marching warm-up training, followed by 20 min of solo air striking training with a 5 min interval. They then engaged in paired confrontation training for 25 min with a 5 min interval, followed by 14 sets of rapid punching for 3 rounds (10 s of interval per set, 1 min of interval per round). This was followed by 3,000 meters of long-distance running training. Finally, all subjects performed 15 min of relaxation air strikes and static stretching to conclude the training. The entire training process utilized a heart rate monitor (Miopod Heart Rate Armband All-in-One Machine, Shuoerda Health Science and Technology Co., Ltd.) to monitor heart rates, and subjects were asked to rate their subjective fatigue on a scale during intervals between each exercise group. The specific training program is detailed in Table 2.

**Table 2:** Exercise fatigue modeling design.

Training content	Training time	Training intensity
Dynamic stretching and marching warm-up training	20 min	Heart rate 100–110 beats/min RPE10-11
Single strike training	20 min	Heart rate 130–140 beats/min RPE13-14
Double match training	25 min	Heart rate 150–160 beats/min RPE15-16
Single 3 rounds of fast punching × 14 sets (10s interval per set, 1 min interval per round)	10 min	Heart rate 160–180 beats/min RPE17-18
3,000 meters running training	15 min	Heart rate 160–180 beats/min RPE17-18

### 2.3 Diagnosis of exercise fatigue

According to the criteria for determining exercise fatigue reported in the literature, athletes’ RPE reached levels of 17–18 after exercise (Lee et al., 2023; Meihua et al., 2023). Urinary protein was negative in the morning of the test day, positive 3 h after exercise, and negative again the following morning.

### 2.4 16S rRNA sequencing

#### 2.4.1 Fecal sample collection

The disposable sterile fecal collection tube (CY-F1-05, purchased from Shenzhen Huachenyang Technology Co., Ltd.) was used for fecal collection. Two to three grams of the subjects’ fecal sample are collected for the first time in the morning by 7 a.m., and the second collection is in the next morning by 7 a.m. after exercise fatigue. Subjects are required to wash their hands and disinfect with 75% alcohol prior to collection. Before collection, place the sterile pad from the disposable sterile fecal sample collection kit at the bottom of the collection basin and use the sterile collection spoon to scoop the unoxidized middle part of the fecal sample, then put it into the collection tube containing sterile DNA preservation solution. Samples are sealed with a sealing film and stored in a 5°C freezer for 4–5 h, then they are transferred to a −80°C refrigerator for storage until testing.

#### 2.4.2 DNA extraction and PCR amplification

Genomic DNA of the samples was extracted using CTAB, and the purity and concentration of DNA were assessed by 1% agarose gel electrophoresis. Samples were diluted to 1 ng/μl using sterile water. PCR amplification of the V3 + V4 variable region was performed using the 341F (5’-CCTAYGGGRBGCASCAG-3′) and 806R (5’-GGACTACNNGGGTATCTAAT-3′) primers.

#### 2.4.3 Purification and mixing of PCR products

PCR products were mixed in aliquots according to their concentration, thoroughly mixed, and purified by agarose gel electrophoresis using 1xTAE at 2% concentration. The target bands were recovered using Universal DNA (TianGen, China).

#### 2.4.4 Library construction and on-board sequencing

The library was constructed using the NEB Next^®^ Ultra DNA Library Prep Kit and quantified by Q-PCR using Agilent 5,400. After qualification, the library was sequenced using the Illumina sequencing platform.

### 2.5 Quality control

Training for subjects and investigators was conducted during the trial period to reduce contamination and DNA oxidation during collection. Urine protein testing was repeated three times to ensure the accuracy of fatigue judgments. Blood collection and preservation were performed by medical professionals. Equipment quality control during the trials was carried out to ensure proper instrument operation and reduce systematic errors.

### 2.6 Bioinformatic analysis and statistics

Based on the default parameters, the DADA2 plugin in the Qiime2 process was used to denoise the optimized sequence after QC splicing. Sequences following DADA2 denoising are often referred to as ASVs. Remove all samples from the annotated mitochondrial sequence. In order to minimize the impact of sequencing depth on subsequent alpha diversity and beta diversity data analysis, the number of sequences in all samples was flattened to 20,000, and the average sequence coverage of each sample could still reach 99.09%. Species taxonomic analysis of ASVs was performed using the Naive Bayes in Qiime2 classifier based on the SILVA 16S rRNA gene database (v_138). Species composition and abundance analyses were performed using R statistical software to conduct *α*-diversity analysis, *β*-diversity analysis, and within-group significance species difference analysis to identify differences in species composition among samples from each group (Rohart et al., 2017).

## 3 Results

### 3.1 Exercise fatigue molding

In this experiment, the average level of creatine kinase in the blood of all subjects increased from 257.4 ± 156.9 U/L to 410.2 ± 84.1 U/L (*p* < 0.01). Meanwhile, the mean value of RPE (The Borg Rating of Perceived Exertion) in box athletes post-exercise was 18.12 ± 0.55.

### 3.2 Data quality

**Figure 2:**
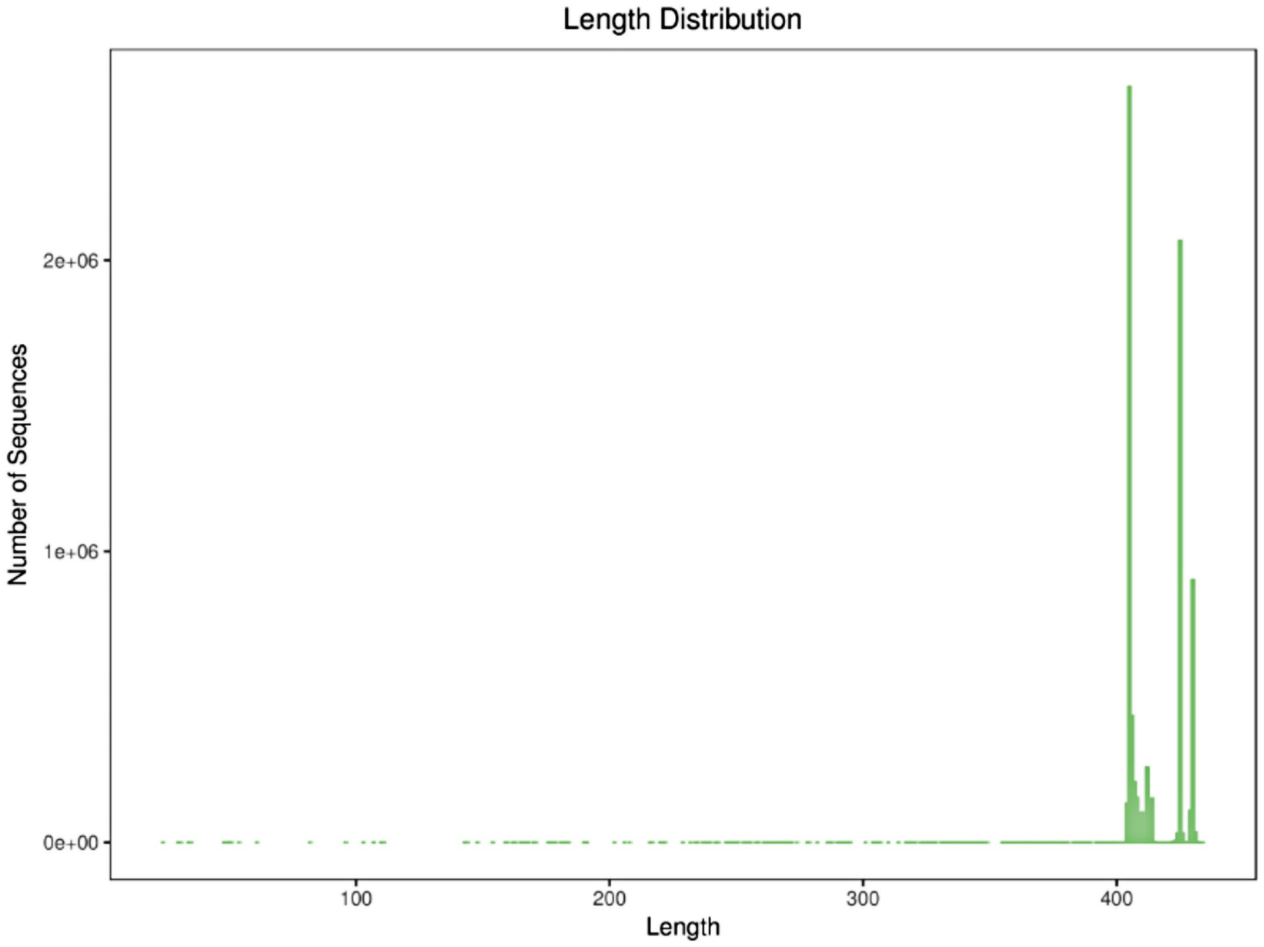
Sequence length distribution.

As a landmark molecular marker in prokaryotic phylogeny, the 16S rRNA gene is widely used in bacterial taxonomic identification and diversity analysis because its sequence has the structural characteristics of both conserved and variable regions. Its conserved regions can be used to design universal primers, while variable regions provide species discrimination information. In the study of human gut microbiota, paired-end sequencing targeting the V3–V4 region has become a standardized protocol for amplicon sequencing, which can balance the taxonomic resolution and sequencing read length limitations (Manos, 2022). This study utilized the Illumina HiSeq sequencing platform to analyze 34 fecal samples collected before and after exercise fatigue. The length distribution of high-quality sequences contained in all samples was counted by 16S rRNA high-throughput sequencing, and it was found that most samples’ sequencing lengths fell within the range of 300–400 bp (base pairs), indicating good stability and sequencing depth, as shown in Figure 2.

### 3.3 ASV cluster analysis

**Figure 3:**
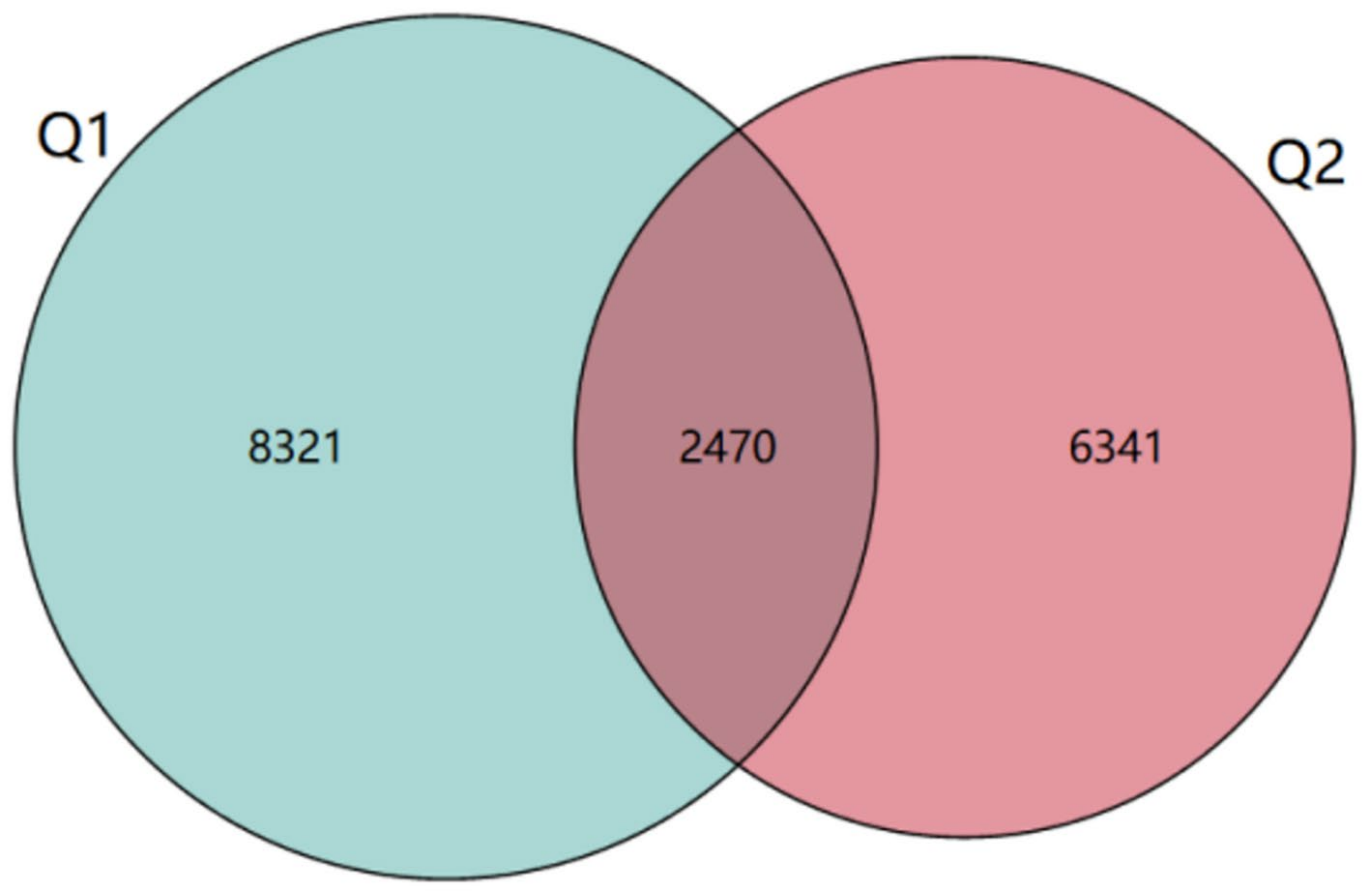
ASV Venn diagram.

ASV is a high-accuracy method for modeling and identifying DNA sequence changes, commonly used to quantify compositional differences between different samples (Suez et al., 2022). In this study, after sequencing, denoising, and merging of 34 samples, a total of 17,132 valid ASV sequences were generated. The ASV sequences before and after exercise fatigue were demonstrated using Venn diagrams, visualizing the similarity of species composition and overlap between samples before and after exercise fatigue. The ASV sequences common and unique to each group were visualized using Venn diagrams. As shown in Figure 3, 2,470 ASV sequences were shared between pre-exercise fatigue and post-exercise fatigue, 8,321 ASV sequences were unique to pre-exercise fatigue, and 6,341 ASV sequences were unique to post-exercise fatigue.

### 3.4 Comparison of alpha diversity of gut microbiota before and after exercise fatigue

**Figure 4:**
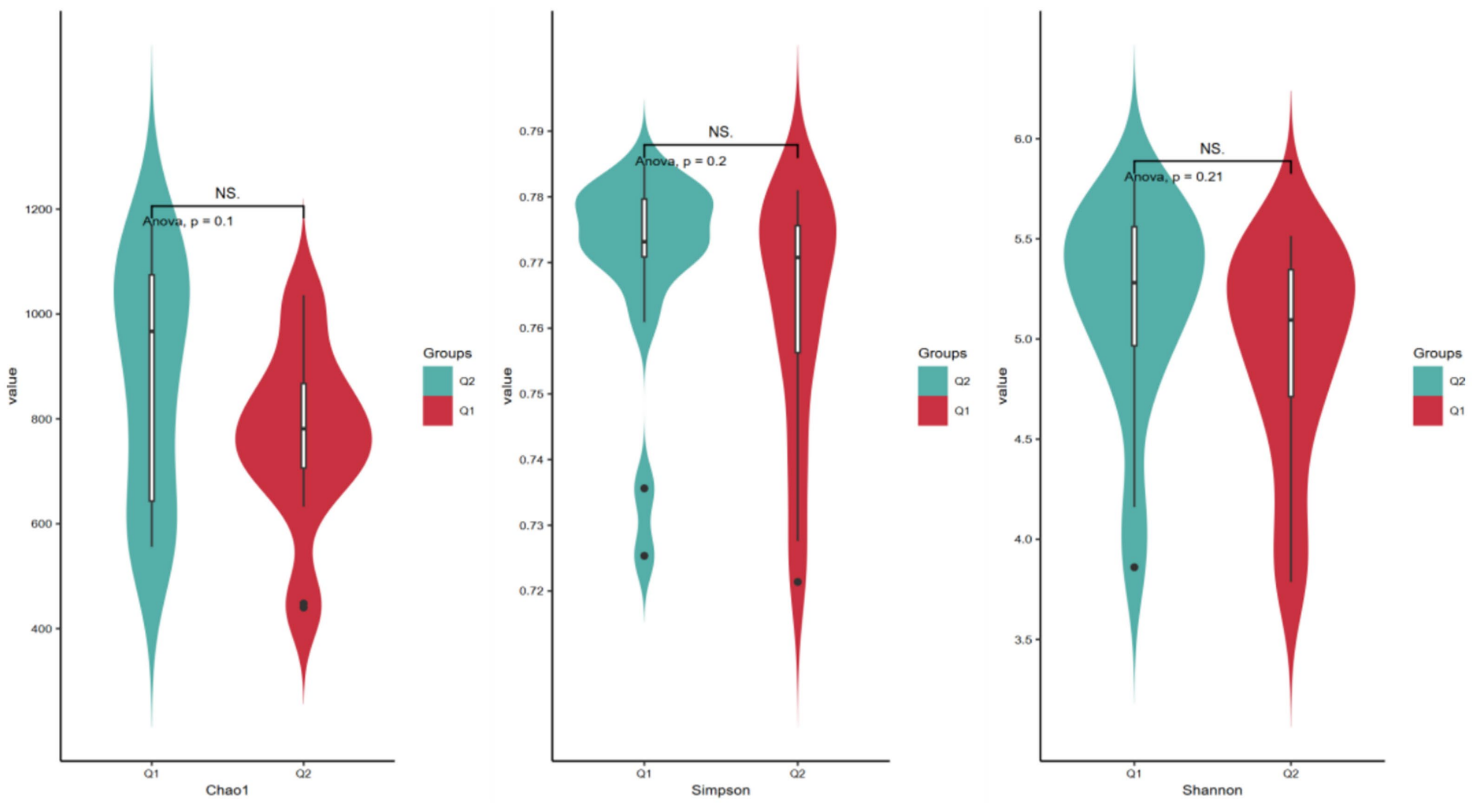
Histogram of the *α* diversity of gut microbiota before and after exercise fatigue.

Alpha diversity refers to the diversity in the distribution of the number and frequency of species at the species level within a particular habitat or microbial community, encompassing both species richness and evenness. The Chao1 index, Shannon index, and Simpson index are commonly employed to reflect species differences in overall composition and abundance (Samara et al., 2022). Specifically, the Chao1 index serves to estimate microbial community species richness, particularly valuing the assessment of potentially unobserved species within a sample. Meanwhile, the Shannon diversity index integrates species richness and evenness to characterize community diversity, demonstrating heightened sensitivity to the distribution patterns of low-abundance species. In contrast, the Simpson index quantifies the concentration degree of dominant species to elucidate community structural characteristics, with its calculation methodology prioritizing the contribution weight of high-abundance species. As presented in Table 3 and Figure 4, prior to and following exercise-induced fatigue, the Chao1 index exhibited a decline from 879.98 to 766.29, the Shannon index decreased from 0.769 to 0.761, and the Simpson index dropped from 5.17 to 4.93. Nevertheless, paired-sample *t*-test outcomes revealed no statistically significant differences (*p* > 0.05).

**Table 3:** Changes in the α diversity of gut microbiota before and after exercise fatigue.

Alpha diversity	Before exercise fatigue	After exercise fatigue	*p*-value
Chao1 index	879.98 ± 217.55	766.29 ± 159.11	0.1
Shannon index	0.769 ± 0.018	0.761 ± 0.015	0.2
Simpson index	5.17 ± 0.51	4.93 ± 0.52	0.21

### 3.5 Comparison of beta diversity of gut microbiota before and after exercise fatigue

**Figure 5:**
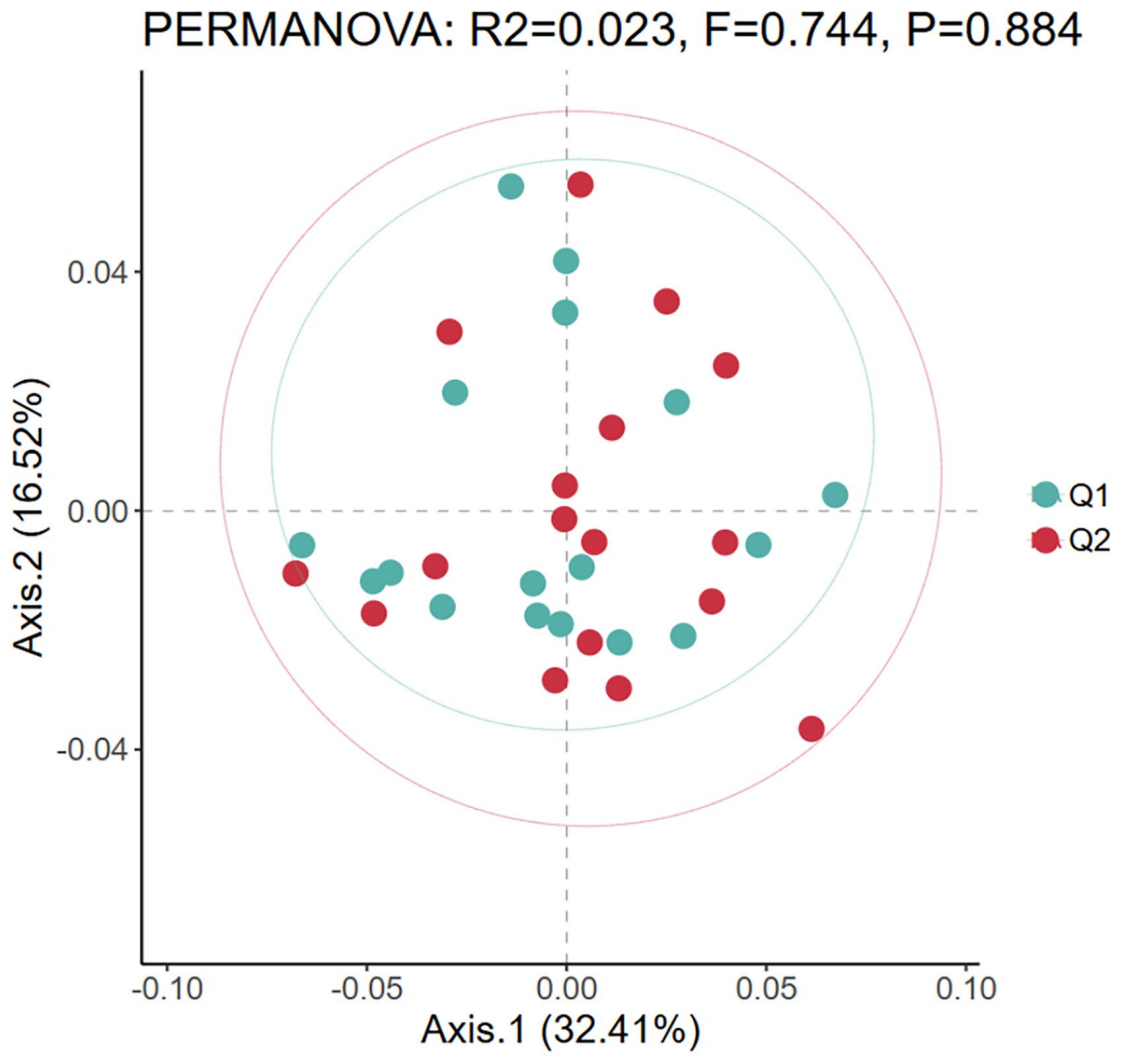
PCoA diagram of beta diversity before and after exercise fatigue.

Beta diversity refers to the dissimilarity of species composition between different microbial communities under environmental gradients or the rate of species turnover along the environmental gradients, mainly reflecting the diversity of species communities and the degree of isolation between species in the region (Zhang et al., 2020). In this study, PCoA analysis based on Bray-Curtis distance was used to visualize differences in gut microbiota between pre-exercise fatigue (Q1) and post-exercise fatigue (Q2). Each point represents a sample; the closer the sample distance, the more compact the microbiota structure. A Bray-Curtis distance based Adonis analysis was conducted, which revealed that no statistically significant difference in *β*-diversity was observed between the pre-exercise fatigue and post-exercise fatigue states (*p = 0.884*) (Figure 5).

### 3.6 Analysis of differences in relative abundance of gut microbiota before and after exercise fatigue

**Figure 6:**
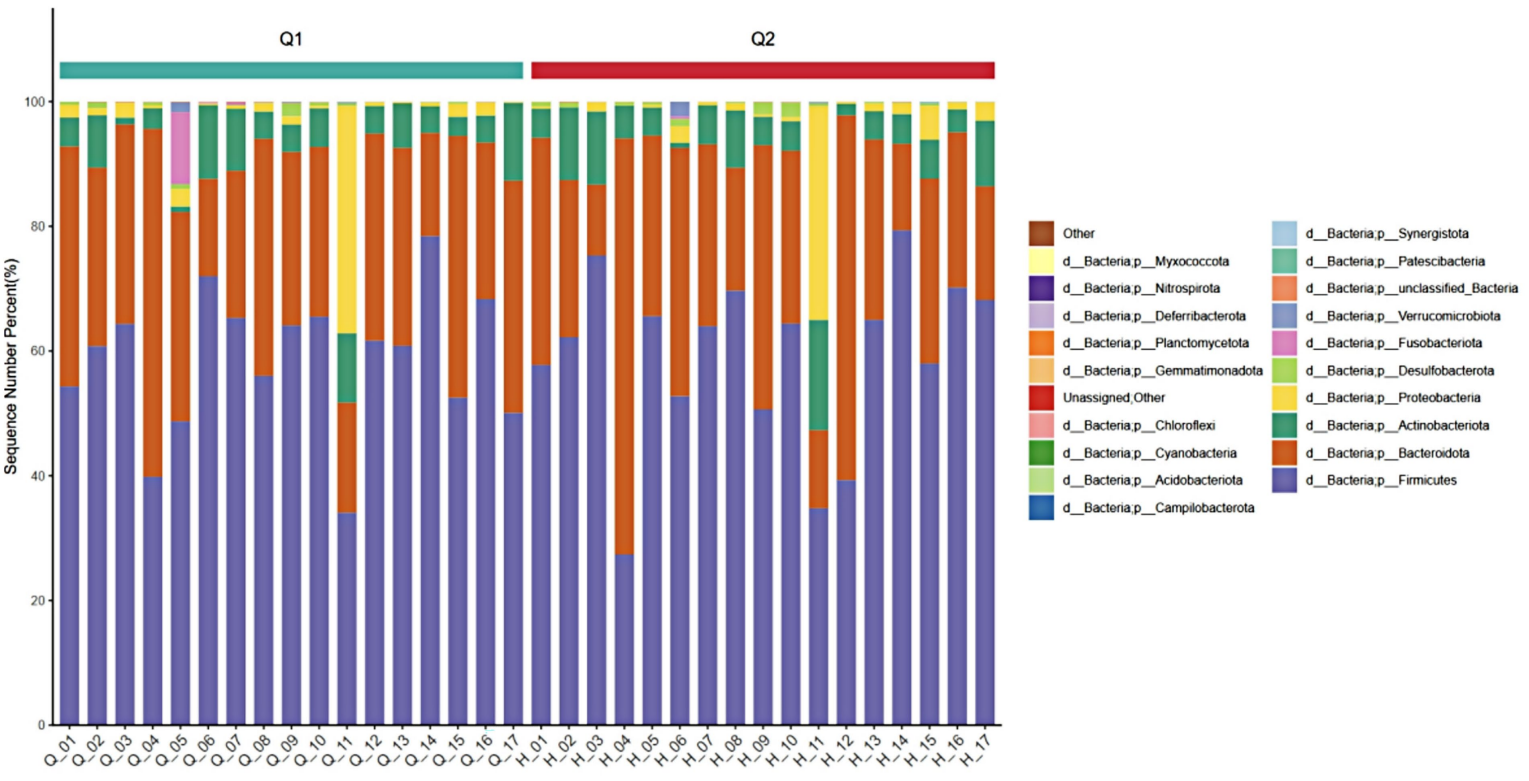
Stacked diagram of before and after exercise fatigue at the phylum level.

At the phylum level, four phyla with an average relative abundance of ≥0.01 in each group were selected, which were *Firmicutes*, *Bacteroidetes*, *Actinobacteria*, and *Proteobacteria*. As shown in Figure 6, the relative abundance of *Firmicutes* (from 59.97 to 59.09%) decreased, the relative abundance of *Bacteroidetes* (from 31.69 to 30.23%) decreased, the relative abundance of *Actinomycetes* (from 5.92 to 6.62%) increased, and the relative abundance of *Proteobacteria* (from 0.11 to 0.056%) increased. The results indicated that, compared to pre-exercise, post-exercise caused the relative abundance of the intestinal flora of the athletes decreased in the phyla *Firmicutes* and *Bacteroidetes*, while the abundance of other phyla increased.

### 3.7 Analysis of differences in specific microbiota before and after exercise fatigue

**Figure 7:**
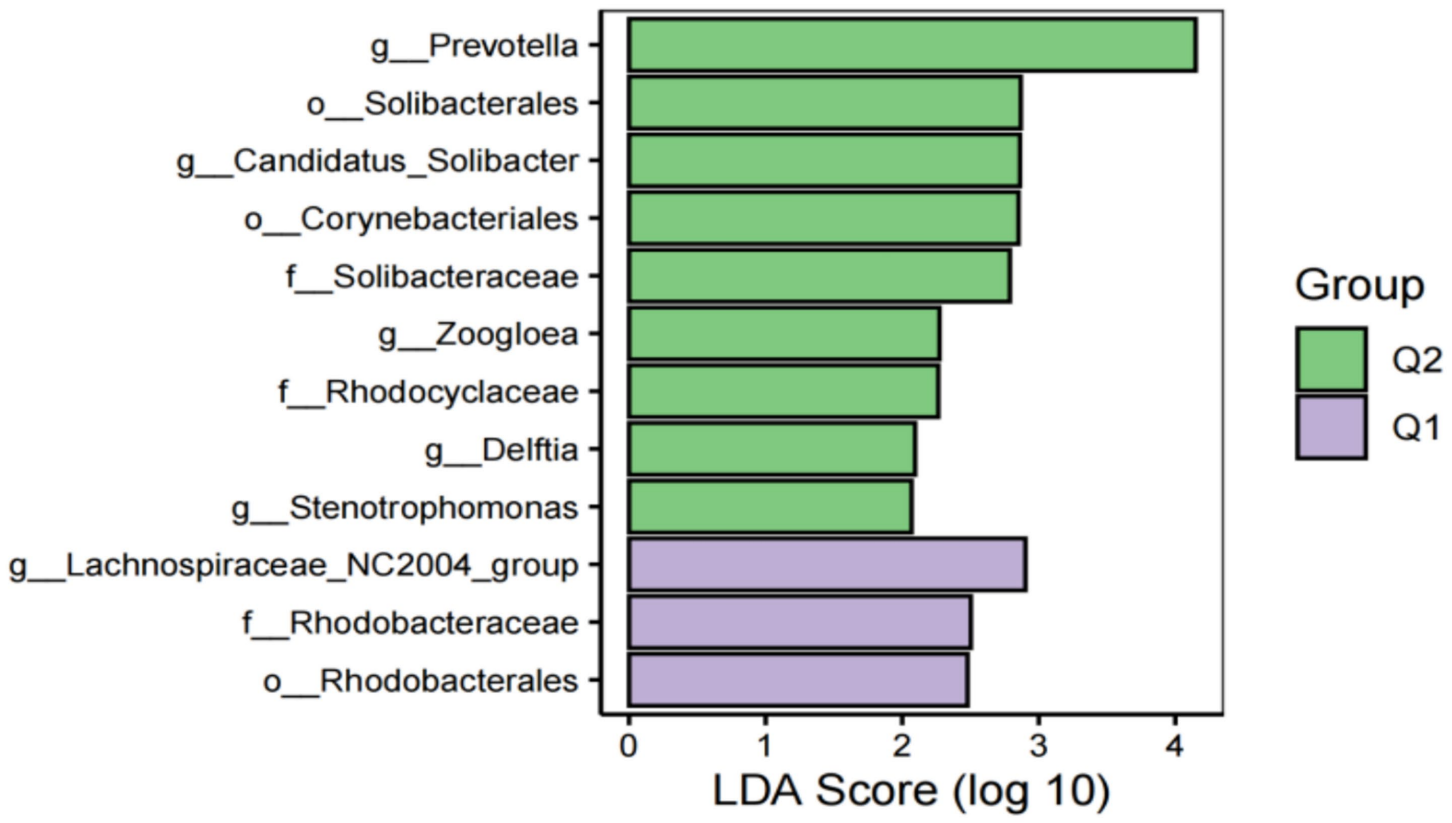
Histogram of LDA effect values of marker species before and after fatigue.

**Figure 8:**
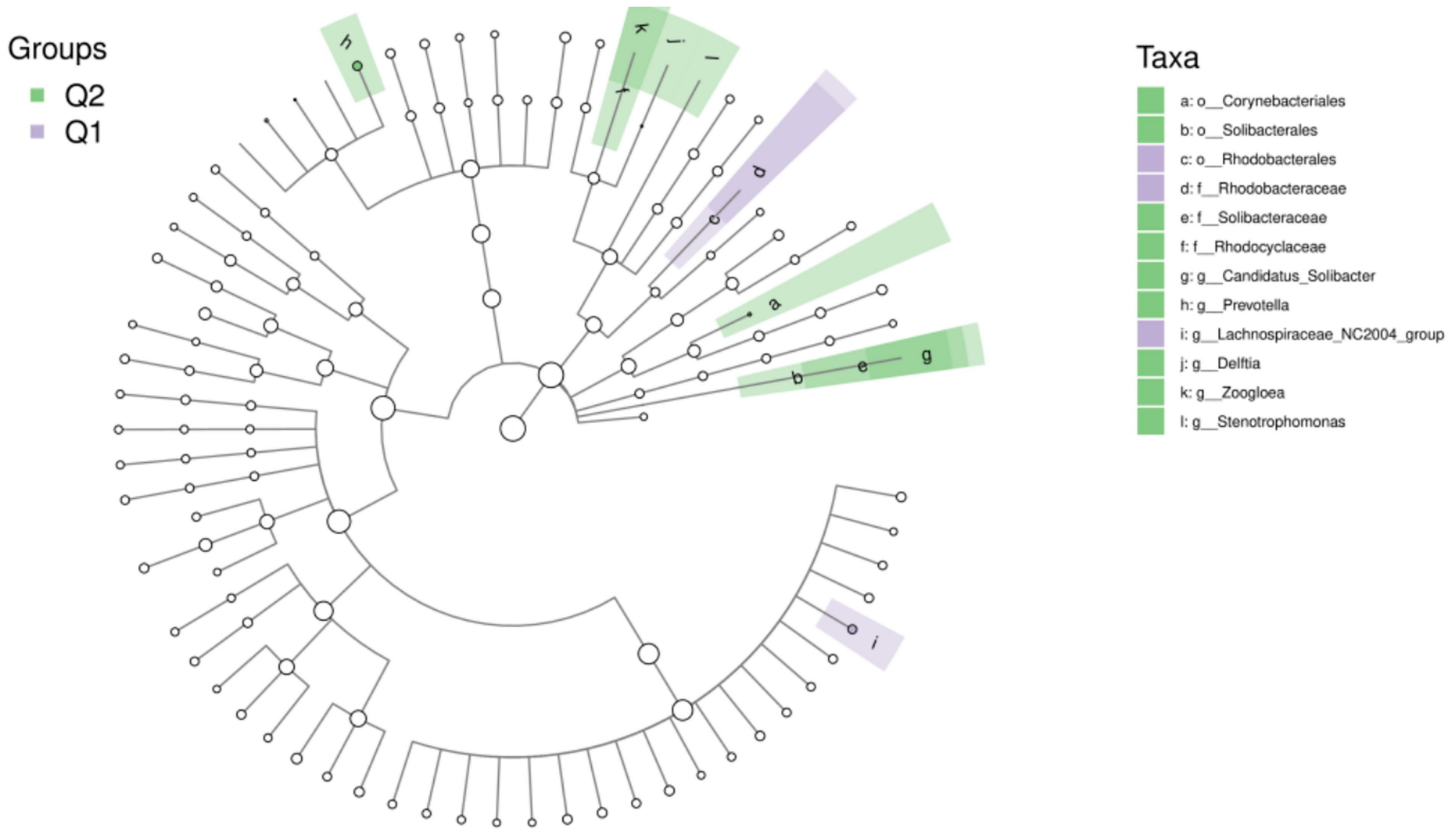
Taxon of differences between LEfSe microbiota groups before and after exercise fatigue.

To further explore the differences in host gut microbiota before and after exercise fatigue and to probe for specific microbiota, LEfSe multilevel species difference discriminant analysis was used to screen for biomarkers of important microbiota with an LDA score (log_10_) = 2 as the cutoff value. The results are shown in Figures 7, 8. There were a total of 12 important microbiota with differences (|LDA| > 2, *p* < 0.05), with key phyla including by *Acidobacteriota*, *Bacteroidota*, and *Proteobacteria* at the phylum level. The top three selected groups in relative abundance were *Prevotella* (*p* = 0.025), *Corynebacteriales* (*p* = 0.038), and *Lachnospiraceae* (*p* = 0.004); After exercise fatigue, the relative abundances of *Prevotella*, *Corynebacteriales*, and *Lachnospiraceae* increased from 4.79 to 4.81, 3.18 to 3.20, and 3.56 to 3.74, respectively, compared to pre-exercise levels.

### 3.8 Correlation analysis of differential microbiota and fatigue phenotype

**Figure 9:**
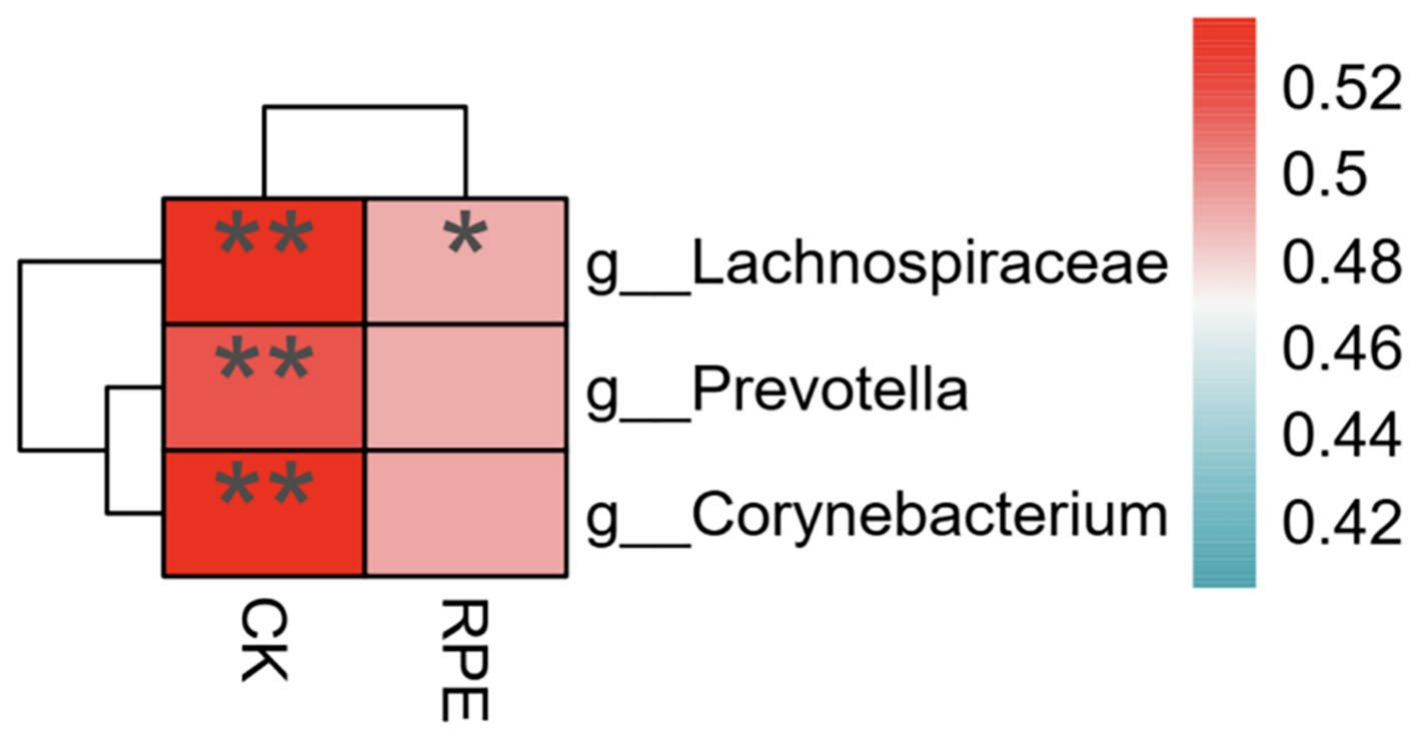
Correlation analysis between differential microbiota and fatigue phenotype after.

The relative abundance value of differential microbiota was > 0.1, and |LDA| > 2; Spearman correlation analysis was used to analyze the correlation between the relative abundance of differential microbiota and the changes in creatine kinase and RPE scale after exercise-induced fatigue, and according to Figure 9, *Prevotella*, *Corynebacteriales*, *Lachnospiraceae* were positively correlated with creatine kinase, and *Lachnospiraceae* was positively correlated with RPE scale.

## 4 Discussion

Since the nineteenth century, scientific research on the mechanisms of exercise fatigue has been deepening, with causes elaborated from various perspectives and hypotheses such as “depletion theory,” “obstruction theory,” and “internal environment homeostasis disruption theory” being recognized by the industry (Meeusen et al., 2021). However, the mechanism of exercise fatigue generation remains unclear. Exercise fatigue is a common and reversible physiological change, serving as an early warning signal for the body to maintain health by reducing exercise load, preventing energy depletion, and preventing exercise injury. Moderate exercise fatigue can promote the storage of energy substances in the host and enhance the recovery of excess energy, enhancing physical fitness and athletic performance (Constantin-Teodosiu and Constantin, 2021). Excessive exercise fatigue can lead to excessive production of oxygen free radicals, triggering endogenous inflammation in the host, suppress immune system, and disrupt the function of the host’s intestinal tract, resulting in fatigue, lack of concentration, diarrhea, and increased risk of upper respiratory infections (Bestwick-Stevenson et al., 2022).

The gut microbiota, a complex community of microorganisms inhabiting the mammalian gut, plays a crucial role in mediating host digestion and absorption, regulating body immunity, participating in material metabolism, and transmitting biological signals (Sabater et al., 2023). Imbalances in gut microbiota are closely related to host fatigue, and the gut microbiota can be used as an important “anchor point” for assessing the health status of the host (Zhang et al., 2024). The gut microbiota of athletes significantly differs from that of sedentary populations, being more diverse and functionally rich, but it can lead to intestinal dysfunction and induce locomotor activity in the fatigued state. The gut microbiota of athletes is significantly different from that of sedentary people, and the diversity and function of athletes’ gut microbiota are richer (Mohr et al., 2020). However, in the state of fatigue, it can lead to intestinal dysfunction, thereby affecting normal training performance and recovery (Mohr et al., 2020).

This study found that the diversity of intestinal microorganisms did not change in acute exercise through tests in the gut microbiota of boxers after exercise fatigue, similar to the findings of Grosicki et al. (2019) and Scheiman et al. (2019). This suggests that the stimulation of the intestinal tract by acute exercise is relatively weak and insufficient to cause changes in the diversity of gut microbiota. However, this study found that after exercise fatigue, the Chao1 index decreased from 879.98 to 766.29, the Shannon index decreased from 0.769 to 0.761, and the Simpson index decreased from 5.17 to 4.93, indicating that the diversity of gut microbiota decreased after fatigue. This may be due to the significant physiological stress on the intestinal tract and the disturbance of the internal environment by strenuous exercise, similar to the findings of Guo et al. (2023) and Hajjar et al. (2021). The majority of Gram-positive bacteria in the Phylum *Firmicutes* indirectly regulate hunger and satiety through metabolites (e.g., short-chain fatty acids) and participate in host energy metabolism and biosynthesis (Stojanov et al., 2020), while the majority of Gram-negative bacteria in the Phylum *Bacteroides* play a role in intestinal ecology through enzymatic breakdown of complex carbohydrates, activation of T-cell-mediated responses, prevention of specific inflammatory responses, and positive regulation of the intestinal internal environment (Gui et al., 2021). The ratio between Phylum *Firmicutes* and Phylum *Bacteroides* (F/B) is related to the maintenance of homeostasis in the body, and changes in this ratio may lead to various pathologies, such as obesity, inflammatory bowel disease, and diabetes mellitus (Pickard et al., 2017). It has been pointed out that inflammatory bowel inflammation can be induced and exacerbated when the F/B ratio decreases (Vaiserman et al., 2020). In this study, we observed a decreasing trend in the abundance of Firmicutes and Bacteroidetes after exercise fatigue compared to pre-exercise levels, suggesting that strenuous exercise leads to dysbiosis in the gut microbiota, affecting the intestinal barrier and function, aggravating endogenous inflammation, and leading to weakening of host intestinal digestion and absorption, which in turn aggravates the accumulation of fatigue.

Related studies have found that *Prevotella* possesses enzymes and gene clusters necessary for fermentation and utilization of complex polysaccharides and may interact with other microbiota to induce visceral hypersensitivity and exacerbate inflammation in the intestinal tract by facilitating carbohydrate fermentation (Randis and Ratner, 2019). Additionally, *Prevotella* contains enzymes that play an important role in mucin degradation, which may lead to increased intestinal permeability. In this study, the relative abundance of Prevotella before and after exercise fatigue was analyzed using the LEfSe multilevel species difference discrimination method (Segui-Perez et al., 2024). The relative abundance value of *Prevotella* increased from 4.79 to 4.81, indicating that exercise fatigue increased the abundance of *Prevotella*. This suggests that fatigue caused by high-intensity and strenuous exercise may dysregulate *Prevotella* in the intestines, affecting intestinal barriers and functions and exacerbating the dysfunctions of the internal environment of the organism. *Corynebacterium* is a Gram-positive bacillus and an opportunistic pathogen; when the host is immunocompromised, it causes endogenous infection by releasing exotoxins, causing systemic toxicity symptoms (Sergeev et al., 2020). It has been suggested that the increase of *Corynebacterium* may be related to host immunocompromise (Liu et al., 2024). In this study, the relative abundance of Corynebacterium increased from 3.18 to 3.20 after exercise fatigue compared to pre-exercise levels, indicating that fatigue causes a decrease in the host’s immune function, and its *Corynebacterium* has a dysfunctional relationship with the rest of the microbiota, which in turn affects the host’s intestinal immune function and aggravates the immune hypofunction caused by exercise fatigue.

In summary, exercise-induced fatigue was associated with an increment in the relative abundance of *Prevotella*, *Corynebacterium*, and *Lachnospiraceae*.

## Study limitations and alternatives

First, the small sample size is mainly to provide an effective methodology and provide a research basis for future large-sample, multicenter trials. second, there is no blinding, because scientific ethics requires that subjects must be informed about the experiment before it is conducted, which is unavoidable; Thirdly, the research funding of this topic is limited, the sample screening requirements are high, and the number of subjects who have reached the diagnosis of exercise-induced fatigue is small, and the included subjects are required to eat in the same canteen as much as possible, and professional nutritionists will make nutritious meals for them to minimize the impact of diet on intestinal flora.

## Data Availability

The datasets presented in this study can be found in online repositories. The names of the repository/repositories and accession number(s) can be found in the article/supplementary material.
